# Factors Associated with Growth Retardation in Children Suffering from Sickle Cell Anemia: First Report from Central Africa

**DOI:** 10.1155/2017/7916348

**Published:** 2017-01-30

**Authors:** Aimé Lukusa Kazadi, René Makuala Ngiyulu, Jean Lambert Gini-Ehungu, Jean Marie Mbuyi-Muamba, Michel Ntetani Aloni

**Affiliations:** ^1^Division of Paediatric Haemato-Oncology and Nephrology, Department of Paediatrics, University Hospital of Kinshasa, Faculty of Medicine, University of Kinshasa, Kinshasa, Democratic Republic of the Congo; ^2^Department of Internal Medicine, Faculty of Medicine, University of Kinshasa, Kinshasa, Democratic Republic of the Congo

## Abstract

*Background*. The aim of this study was to investigate and determine the risk factors associated with poor growth among SCA children.* Methods.* A cross-sectional study was conducted in Kinshasa, the capital's country. The nutritional status was assessed using the Z scores of the anthropometric indices.* Results.* We gathered data on the 256 patients, 138 females (53.9%), who entered the study. The mean age at presentation was 8.4 ± 4.9 years of age. Underweight, stunting, and wasting were found, respectively, in 47.7%, 10.5%, and 50.3% of SCA children. A history of hand-foot syndrome, more than 3 blood transfusions, being less than 12 months of age when receiving the first transfusion, more than two severe sickle crises per year, a medical history of severe infections, and the presence of hepatomegaly were associated with poor growth. When comparing sickle cell patients under 12 years of age (*n* = 159) to a group of 296 age-matched children with normal Hb-AA, a significantly higher proportion of subjects with stunting and underweight were found among SCA.* Conclusion.* Nutritional status encountered in Congolese sickle cell children has been described for the first time in this study. A high prevalence of poor growth in SCA children was found in our study.

## 1. Introduction

Sickle cell anaemia (SCA) is the commonest genetic diseases in sub-Saharan Africa [1]. In the Democratic Republic of Congo (DRC), the HBB^*∗*^S allele prevalence in neonates ranges from 0.96% to 1.4%. According to 2010 estimation, the DRC contributed to the global burden of SCA with 39,800 [CI: 32,600–48,800] neonates each year [2].

SCA is an inflammatory disease characterized by chronic haemolysis, vasoocclusive crises, severe infection, and organ damage [1]. It is known that the sickle cell neonates have a normal weight at birth [3, 4]. However, the disease with its attendant increased energy requirements has a negative effect on growth with a slow prepubertal growth and a delayed velocity compared with normal children [5]. Many factors as endocrine and/or metabolic dysfunction, haematological status, and nutritional status may play an important role in growth failure [5, 6].

Great progress has been made in the care of children with SCA in recent decades [7, 8]. Management of this haemoglobinopathy has been changing and is excellent as we have patients with advanced age in developed countries [8, 9]. However, in developing countries such as the DRC, these cures are compromised by financial, human and laboratories resources deficiencies, inconsistent drug supplies, and delayed time of diagnosis [10, 11].

The determinants of low growth are not well understood and are probably due to phenotypic polymorphism due to haplotype, genetics factors, foetal haemoglobin level, specific nutrient deficiencies, and environmental factors [12–14]. Most studies are performed in sickle cell patients living in variable conditions that may affect growth and contribute to the difficulty to understand the mechanisms of growth in this population [15].

In the Democratic Republic of Congo (DRC), there is still very little information on nutritional status of children with SCA, because most provinces of this country lack paediatricians and vital hospital statistics scarcely contain sur information [16]. Additionally, 80% of the population lives in extreme poverty and more than 70% are estimated to suffer from malnutrition. Furthermore, the severity of the disease added to the poverty living conditions may influence the sickle cell children's growth curve [17]. Despite the high incidence and prevalence of SCA and the risk of failure to thrive in these patients, there was a paucity of studies from Central Africa, highlighting a large knowledge gap for low-resource settings.

It is necessary for health planning to have the main characteristics of children with SCA living in the DRC. This information will give the whole view of patients and may serve to rule out public health and in-hospital politics in our midst.

Thus, the aim of this study was to determine the prevalence and secondly to investigate the risk factors associated with failure to thrive among SCA children living in Kinshasa, the DRC. Our findings were compared to the results of previous studies reported in the literature.

## 2. Methods

This cross-sectional study was conducted in three paediatric health facilities in Kinshasa, namely, Centre de santé Saint Sacrement de Binza (West), the Sickle Cell Center of Yolo (Centre), and Centre de Santé Saint Marc (East). These health facilities provide most of the paediatric sickle cell follow-up in Kinshasa, DRC. These hospitals also provide most of the nonprivate paediatrics beds in Kinshasa for sickle cell patients.

Patients were consecutively selected in the outpatient clinic of the three health institutions. For the growth comparison, each sickle cell child under twelve years of age was matched with one or two control AA children for age, sex, and place of residence.

The following clinical and laboratory information were collected and analyzed: (i) demographic characteristics, (ii) anthropometric parameters, (iii) age at the first pain crisis and at the first blood transfusion, (iv) number of severe sickle cell crises per year, (v) blood transfusion, and (vi) haematologic parameters.

## 3. Data Collection Procedure and Analysis

Height and weight were measured using standardized techniques. Weighing scale was calibrated to zero before taking every measurement. The children were weighed with minimum clothing and without shoes.

Measurement of height was done in a lying position with wooden board for children under 2 years of age and measurement of height for children over 2 years of age was in a standing position in centimeters to the nearest 1 cm.

The nutritional status of the study population was assessed using the Z scores of the anthropometric indices: weight-for-height Z score (WHZ), height-for-age Z score (HAZ), and weight-for-age Z score (WAZ). These indicators were calculated according to the references of the National Centre for Health Statistics/WHO/CDC [18]. Abnormal status is defined as having an indicator Z score less than −2.0. WHZ determined the severity of wasting, HAZ the severity of stunting, and WAZ underweight.

At the end, the nutritional status of SCA patients was compared to healthy peers age-matched haemoglobin-AA group. Because sex steroid hormones effects on the growth pattern start on average at 12 [19], we only compare children under 12 years of age for this purpose, in both groups.

Blood samples were collected from all subjects. Sickle cell screening was performed using isoelectric focusing (IEF) technique with the Multiphor II apparatus (GE Healthcare, Little Chalfont, England). The separation of different haemoglobin (F, A, S, and other types of haemoglobin) was obtained after application on thin layer home-made agarose gel containing ampholytes pH 6–8 (ref. 2117–003; Pharmalyte pH 6.7–7.7; GE Healthcare).

## 4. Data Management

The information that was obtained was analyzed using Epi info version 6 (CDC). After data cleaning (control for quality and coherence), they were exported on SPSS 22.0 for further analysis. All data from discrete variables are represented as means ± standard deviation (SD). Frequency of various clinical and laboratory findings are expressed as percentages. The confidence interval at 95% was calculated. Pearson chi-square or Fisher's exact test was used to assess differences in categorical data between groups. The analysis of Student's *t*-test or Mann–Whitney test was used for comparisons of means. The relationship between growth in SCA children and study parameters was assessed using logistic regression models. Odds ratios were provided with their 95% confidence interval (95% CI) and were estimated for the factors that have a significant effect. A *p* value < 0.05 was considered significant.

## 5. Results

### 5.1. Study Population and Baseline Characteristics

We gathered data on the 256 patients, 138 females (53.9%) and 118 males (46.1%), who entered the study. The mean age at presentation was 8.4 ± 4.9 years of age.  Table 1 presents baseline characteristics of all patients.

### 5.2. Risk of Short Stature

Growth in height fell below the 5th percentile in 7.8% (*n* = 20) of sickle cell children. A history of hand-foot syndrome and the number of transfusions of more than 3 per patient were associated with an increased risk of short stature with OR, respectively, 4.3 and 4.8. However, the presence of hepatomegaly was also associated (OR 4.2) but with a nonsignificant CI 95%. All results are shown in Table 2.

### 5.3. Underweight, WAZ

In this series, underweight was found in 47.7% (*n* = 122) of SCA children. The presence of hepatomegaly was weakly associated with an increased risk of wasting.

### 5.4. Stunting TAZ

In this series, stunting was found in 10.5% (*n* = 27) of SCA children. Number of severe sickle cell crises of more than two per year, age at the first blood transfusion less than 12 months, and number of blood transfusions of more than three per patient were associated with an increased risk of stunting (Table 2).

### 5.5. Wasting WHZ

According the WHZ, wasting was found in 50.3% (*n* = 129) of SCA children. A history of hand-foot syndrome is the main factor associated with an increased risk of wasting. Other factors such as history of severe infections and the number of transfusions of more than 3 per patient were weakly associated.

### 5.6. Comparison with Normal Children (Hb-AA)

Among children under 12, a significantly higher proportion (34.9%) of subjects with stunting were children with Hb-SS, compared to 9.8% in children with Hb-AA. Additionally, more than a third (39.8%) of subjects with underweight were children with Hb-SS, compared to 12.2% in children with Hb-AA (Table 3).

In Hb-SS group, the prevalence of wasting (WHZ) tended to be higher than Hb-AA groups. However, there was no statistically significant difference between the two groups (Table 3).

## 6. Discussion

The aim of study is oriented to identify the factors associated with failure to thrive in a paediatric population. In Central Africa, a predominant region of Bantu haplotype, there is anecdotal information on growth in paediatric population suffering from SCA [20]. Our study is the first to investigate and determine the risk factors associated with failure to thrive among SCA children living in Kinshasa, the capital's country of the DRC.

Before going through discussing our results, it appears important to explain difficulties when using growth chart from different populations. Ideally, local charts would give better insight of the real situation and local realities. Unfortunately, to the better of our knowledge, available references for growth studies in our midst are the ones we used here from the National Centre for Health Statistics/WHO/CDC [18]. The advantage would be to have an international standard growth chart which allows comparison of children in different settings around the world.

Growth in height fell below the 5th percentile in 7.8% of sickle cell children. Similar observations on short stature were reported in other worldwide studies where the proportion varies from 5% to 54% [5, 21–24]. These wide variations are probably due to sample size, selection of reference growth data, and environmental and genetics factors which limited comparability between these studies.

A history of hand-foot syndrome, the number of transfusions of more than 3 per patient, and the presence of hepatomegaly were associated with an increased risk of short stature. These trends are in accordance with the results found in the literature [25].

In this series, underweight was found in 47.7% of SCA children. Similar observations were reported in other worldwide studies [24, 26–28]. The presence of hepatomegaly was weakly associated with an increased risk of wasting. This observation is probably due to the predominance of SCA without *αα*+thalassemia in Central Africa et the severity of this phenotype [29, 30]. In contrast to our findings, Al-Saqladi et al. found an association of low height-for-age Z score with increased age [22]. These differences between our study and the Yemen study presumably arise from differences in environmental and genetic factors that influence growth of sickle cell children

In this series, stunting was found in 10.5% (*n* = 27) of SCA children. These results are in consonance with previous studies where the prevalence varies from 16.4% to 43% [20, 24, 27, 31]. Number of severe sickle cell crises of more than two per year, age at the first blood transfusion less than 12 months, and number of blood transfusions of more than three per patient were associated with an increased risk of stunting. This may find explanation as these factors provide a trend of the severity of the disease. The severity of the clinical manifestations of the disease increases the energy expenditure and decreases the calorie intake [32]. The results found in this cohort confirm and extend the findings of several studies [20, 25, 33]. In contrast, Al-Saqladi et al. found an association of low height-for-age Z score with male gender in their series [22]. These differences between our study and the Yemen study presumably arise from differences in environmental and genetic factors that influence growth of sickle cell children.

According the WHZ, wasting was found in half of our of SCA children (50.3%). This prevalence is higher than the results reported by Henderson et al. in USA where 11% of sickle cell children were affected [31]. These differences may be due to selection of reference growth data and environmental and genetics factors. Only a history of hand-foot syndrome is strongly associated with an increased risk of wasting, while severe infections and the number of transfusions of more than 3 per patient were weakly associated. This is in accordance with those reported by other authors [24, 25]. In contrast, Al-Saqladi et al. in Yemen found an association of low weight-for-height Z score with increased age [22]. These differences between our study and the US study presumably arise from differences in environmental and genetic factors that influence growth of sickle cell children.

In this series, age, gender, increased volume of tonsil, age at the first transfusion, splenic sequestration, WBCs, and platelets levels were not associated with poor growth as described by other authors [31]. In contrast, these parameters were found to be associated with poor growth in previous worldwide studies [3, 20]. These differences between these studies presumably arise from differences in environmental and genetic factors that influence growth of sickle cell children.

From these findings, only little explanation may be advanced and speculated. Underweight and wasting are probably due to phenotypic polymorphism due to haplotype, genetics factors, fetal haemoglobin level, specific nutrient deficiencies, and environmental factors [12–14]. Indeed, the Bantu haplotype is predominant and the Congolese SCA patients displayed low levels of fetal haemoglobin (HbF) and F-cells that contribute to the severity of SCA [34]. To the best of our knowledge, it appears that factors associated with growth in SCA children are those that characterize more severe disease.

A significantly higher proportion of subjects with stunting were children with Hb-SS, compared to 9.8% in children with Hb-AA. The HAZ (stunting) scores were significantly more common among SCD children than controls, in previous worldwide studies [20, 35, 36]. In contrast, Barden et al. in Nigeria found no significant difference between normal and sickle cell children [36]. These differences between our study and the US study presumably arise from differences in environmental and genetic factors that influence growth of sickle cell children. Limitation from our study may arise in the fact that we did not assess nutritional intake of each patient as this was not the aim of the study. Mandese et al. showed that inadequate nutritional intake, weight, and Body Mass Index have significant impact on SCA severity indices such as number of hospital admissions per year, days of hospital admission per year, and mean haemoglobin F [32].

A significantly higher proportion (39.6%) of subjects being underweight were children with Hb-SS, compared to (12.2%) in children with Hb-AA. This observation is comparable with previous studies in African countries [35, 36]. In Hb-SS group, the prevalence of wasting (WH-Z score) tended to be higher than Hb-AA groups. However, there was no statistically significant difference between the two groups. This observation is also reported by previous studies in African and worldwide countries [35–38]. In USA, Malinauskas et al. found that WHZ did not differ from national reference data [25]. However, the sample size of the US study was limited to children under 6 years of age. This pattern is understandable because early event has by itself less impact on a chronic disease, and growth processes into phases which are influenced by different hormones [19].

The present study had some limitations due to its hospital-based cross-sectional design and a retrospective chart review. One additional limitation was biological measurements to perform during the study. Also, as Kinshasa accounts only for 15% of the DRC population, this study may not necessarily reflect the overview of the whole country. But this should be the general trend since our patients came from all different socioeconomic areas. Despite these limitations, these data provide insights into the relationship between poor growth and SCA in our midst.

## 7. Conclusion

Nutritional status encountered in Congolese sickle cell children has been described for the first time in this study. A high prevalence of poor growth in SCA children was found and was associated with more severe disease estimated by several clinical characteristics in our study. Stunting is more common compared to underweight in SCA children. These results reflect the chronicity of SCA. Additionally, Congolese SCA children are more underweight, wasted, and stunted than the Hb-AA children. The high prevalence of sickle cell anaemia in Sub-Saharan Africa underlines the need for screening of all SCA children to identify patients with the risk of developing poor growth and provide early management. Furthermore, our results strongly suggest that more research in our midst would provide valuable insights into the pathogenesis of poor growth.

## Figures and Tables

**Table 1:** Characteristics of the study population.

Parameters	Patients (*n* = 256)
Age, years	
Mean (SD)	8.4 ± 4.9
Age distribution, years	
<4 years, *n* (%)	(31.6)
5–9 years, *n* (%)	(32.5)
10–14 years, *n* (%)	(25)
≥15 years, *n* (%)	(10.9)
Gender	
Male, *n* (%)	118 (46.1)
Female, *n* (%)	138 (53.9)
Anthropometrics parameters	
Mean weight (kg) (range)	20.6 ± 9.8 (7–62)
Mean height (cm) (range)	115 ± 24 (63–172)
Mean BMI, (range)^*∗*^	17.0 ± 1.8 (10.9–22.9)
Clinical findings	
Hepatomegaly, *n* (%)	136 (53.1)
Splenomegaly, *n* (%)	109 (41.7)
Sickle cell crises	
Haemolysis, *n* (%)	136 (53.1)
Severe pain crisis, *n* (%)	170 (66.4)
Hand-foot syndrome, *n* (%)	85 (33.2)
Severe infection, *n* (%)	45 (17.6)
splenic sequestration, *n* (%)	19 (7.4)
Mean age at the first pain crisis (range), months	18.2 ± 15.2 (2–108)
Mean age at the first transfusion (range), months	29.2 ± 27.6 (2–132)
Number of severe pain crises/year, *n* (range)	3.5 ± 2.9 (1–20)
Number of blood transfusions, *n* (range)	4.1 ± 3.2 (0–30)
Laboratory features	
Mean Hb (g/dl) (range)^*∗*^	7.4 ± 1.5 (4.3–11)
Mean Ht (%) (range)^*∗*^	23.2 ± 4.5 (12.2–35)
Mean WBCs (10^3^/mm^3^) (range)^*∗*^	14.5 ± 5.4 (4.6–34.2)
Mean platelets (10^3^/mm^3^) (range)	315.5 ± 118.5 (114–582)

^*∗*^BMI, body mass index; Hb, hemoglobin; Ht, hematocrit; WBCs, white blood cells.

**Table 2:** Factors associated with a risk of stunting in the study population.

Variables	OR	CI 95%	*p*
Underweight, WAZ			
*Hepatomegaly*			<**0.05**
Absence	**1**		
Presence	**1.4**	**1**–**1.8**	
TAZ, risk of wasting			
*Number of sickle cell crises/year*			<0.01
≤2	1	—	
>2	3.8	1.5–9.5	
*Age at the first transfusion*			<0.01
>12 months	1	—	
≤12 months	3.3	1.1–9.3	
*Number of blood transfusions*			<0.05
≤3	1	—	
>3	2.5	1.1–6	
Risk of short stature			
*Hand-foot syndrome*			<0.01
Absence	1	—	
Present	4.3	1.3–13.7	
*Number of blood transfusions*			<0.05
≤3	1	—	
>3	4.8	1.1–21.5	
*Hepatomegaly*			<0.05
Absence	1	—	
Presence	4.2	0.94–18.9	
Wasting WHZ			
*Hand-foot syndrome*			<0.01
Absence	1	—	
Present	1.7	1.2–2.5	
*Severe infections*			<0.05
Absence	1	—	
Presence	1.8	1.0–3.1	
*Number of blood transfusions*			<0.05
≤3	1	—	
>3	1.3	1.0–1.7	

**Table 3:** Nutritional status according to Hb status in children under 12 years of age in the study population.

Variables	Hb-SS, *n* = 159	Hb-AA, *n* = 296	*p*
Stunting TAZ, *n* (%)	55 (34.6%)	29 (9.8%)	<0.001
Wasting WHZ, *n* (%)	23 (14.5%)	37 (12.5%)	NS
Underweight WAZ, *n* (%)	63 (39.6%)	36 (12.2%)	<0.001
